# Performance comparison of structured $H_{\infty }$ based looptune and LQR for a 4-DOF robotic manipulator

**DOI:** 10.1371/journal.pone.0266728

**Published:** 2022-04-11

**Authors:** Arafat Asghar, Muhammad Iqbal, Abdul Khaliq, Saif ur Rehman, Jamshed Iqbal

**Affiliations:** 1 High Voltage and Short Circuit Laboratory, National Transmission and Despatch Company (NTDC), Rawat, Islamabad, Pakistan; 2 Department of Computer Science, National University of Technology (NUTECH), Islamabad, Pakistan; 3 Department of Electrical and Computer Engineering, Sir Syed CASE Institute of Technology, Islamabad, Pakistan; 4 Department of Electrical Engineering, Superior University, Lahore, Pakistan; 5 Department of Computer Science and Technology, Faculty of Science and Engineering, University of Hull, Hull, United Kingdom; University of Bradford, UNITED KINGDOM

## Abstract

We explore looptune, a MATLAB-based structured $H_{\infty }$ synthesis technique in the context of robotics. Position control of a 4 Degree of Freedom (DOF) serial robotic manipulator developed using Simulink is the problem under consideration. Three full state feedback control systems were developed, analyzed and compared for both steady-state and transient performance using the Linear Quadratic Regulator (LQR) and looptune. Initially, a single gain feedback controller was synthesized using LQR. This system was then modified by augmenting the state feedback controller with Proportional Integral (PI) and Integral regulators, thereby creating a second and third control system respectively. In both the second and third control systems, the LQR synthesized gain and additional gains were further tuned using looptune to achieve improvement in performance. The second and third systems were also compared in terms of tracking a time-dependent trajectory. Finally, the LQR and looptune synthesized controllers were tested for robustness by simultaneously increasing the mass of each manipulator link. In comparison to LQR, the second system consisting of Single Input Single Output (SISO) PI controllers and the state feedback matrix succeeded in meeting the control objectives in terms of performance, optimality, trajectory tracking, and robustness. The third system did not improve performance in contrast to LQR, but still showed robustness under mass variation. In conclusion, our results have shown looptune to have a comparatively better performance over LQR thereby highlighting its promising potential for future emerging control system applications.

## 1 Introduction

Current research in robotics is focused on devising novel solutions for a variety of control problems [1] such as executing motion in deformable and uneven spaces, gripping delicate and fragile objects, performing efficient supervisory control for swarm robots etc. These tasks become critically important in the context of human-robot interaction [2]. “Anthropocentric” or “human-friendly” intelligent robotic systems are gradually becoming ubiquitous. However, researchers are confronted by the highly nonlinear, random, and time-varying nature of the control problems [3, 4]. As reported in several works [2, 5–15], adaptive, intelligent and robust control techniques are one of the many strategies having potential to overcome the above-mentioned problems.

Adaptive control algorithms incorporate parameter estimation in one form or the other [16] and is further classified into conventional and robust categories [17]. Although adaptive control demonstrably achieves continuous improvement in tracking error, assumptions on structure and uncertainty size are made in most of the methods. Also, several studies have proven “model-free” or no “prior assumptions on the plant” controllers to exhibit poor performance [18–21]. Intelligent control is the application of artificial or computer-aided intelligence techniques such as neural networks, evolutionary computation, fuzzy logic [22], machine or reinforcement learning to (usually) complex and non-trivial dynamical systems [23, 24]. In [25], a full-state output-feedback adaptive fuzzy controller has been devised with with output constraint. Likewise, an adaptive impedance-based control strategy using neural networks was developed to tackle dynamical uncertainties dynamics for a trajectory tracking problem [26]. Although the future of intelligent control seems promising [27, 28], the cost and failure rate associated with implementing such control schemes is higher in comparison to conventional schemes, especially for complex systems. Moreover, artificial neural network-based control designs are mostly simulation-based with slight evidence of practical implementation [2].

In robust control, a pre-designed static or fixed structure controller compensates for bounded parametric uncertainties or disturbances and therefore does not require online tuning [29, 30]. Thus, robust control offers better performance in terms of responsiveness [31] and practical realization [32] in comparison to adaptive control. An overview of robust control for robotic manipulators has been presented in [33–35]. $H_{\infty }$ control is a well-known robust control technique and has been applied extensively on a variety of rigid and flexible robotic models. Control design for a quadcopter has been developed by integrating nonlinear $H_{\infty }$ and Model Predictive Control [36] and in [37] by using integral sliding mode control. A robust regulator was designed for a 2 Degree Of Freedom (DOF) platform using mixed $H_{2}/H_{\infty }$ synthesis and considering pole-placement constraints in a linear matrix inequality framework [38]. $H_{\infty }$ regulation using local gravitational force compensation for a 3-DOF robotic manipulator was designed in [39]. In [40], a finite-time globally stable $H_{\infty }$ controller was designed without solving for Hamilton-Jacobi equation or Algebraic Riccati equation by using the backstepping method. Luo et al. devised $H_{\infty }$ control for 2-Dimensional (2D) Takagi–Sugeno fuzzy systems characterized by time-delays and missing measurements within a second Fornasini–Machesini local state-space framework [41]. Similarly, a novel 2D sensor state estimator guaranteeing *l*_2_-*l*_∞_ disturbance attenuation and power bound constraints has been developed in [42]. In [43], a novel nonlinear $H_{\infty }$ controller has been designed for a multi-DOF robotic arm using the concept of min-max differential games. An adaptive fuzzy neural network controller based on impedance learning was implemented for a constrained 3-DOF robot in [44]. The idea of inverse optimal Proportional Integral Derivative (PID) control combined with Feed-Forward control in an $H_{\infty }$ framework has been considered in [45]. Finally, $H_{\infty }$ control has been designed for distributed/decentralized control using multiple industrial manipulators [46].

In all of the methods described above, a centralized and full-order controller is obtained without the possibility of enforcing structure. The controllers are synthesized by either Semi Definite Programming (SDP) [47], Algebraic Riccati Equations [48] or bounded real lemmas involving Lyapunov variables. Structural constraints cause Bilinear Matrix Inequalities (BMIs) to be introduced into such $H_{\infty }$ techniques and make them non-convex. Optimization programs of moderate size have been proven to experience numerical complications for such BMIs [49].

The development of novel nonsmooth optimization techniques has allowed for structured $H_{\infty }$ synthesis to be carried out under structural constraints [49, 50]. These algorithms were finally incorporated in MATLAB [51–53] as hinfstruct and looptune commands in the Robust Control Toolbox. While hinfstruct has been successfully applied in several control studies [54–63], it requires the design requirements to be specified in terms of a well-posed $H_{\infty }$ optimization problem. However, strong scaling and tight coupling in multi-loop control systems may lead to ill-posing of the $H_{\infty }$ problem. Erroneous results may also arise due to cross-coupling between feedback loops when considering stability margins of each loop separately [64, 65]. Finally, in the studies described above no comparison of hinfstruct with existing control techniques in literature or improvement thereof has been considered.

On the other hand, the looptune command allows the user to specify the $H_{\infty }$ problem in terms of generalized high-level requirements, which lead to satisfactory results despite the problems described above [51, 53]. The looptune command has been used to tune a 2-DOF PID controller which is then augmented with a reset controller for a 4-DOF robotic arm [66]. In [67], adaptive control is augmented with a looptune/hinfstruct synthesized robust controller. Zhao et al. developed a multi-variable robust controller for an electrified turbocharger using looptune [68]. Finally, in [69] looptune has been used to develop an outer control loop for a flexible x-hale aircraft, in conjunction with Gain Scheduling [70].

This study has been specifically conducted to assess the advantage of the novel MATLAB command looptune. For this purpose, comparison with a well-known optimal control technique, namely the Linear Quadratic Regulator (LQR) is carried out. The control problem under consideration is position control of a 4-DOF robotic arm developed in SimMechanics/Simulink. With LQR, only a single gain can be synthesized for state feedback control. Determination of the optimum solution to the Algebraic Riccati Equation in LQR is often a tedious and time-consuming process. Finally, LQR does not provide robustness against uncertainties or disturbances. On the other hand, looptune allows minimization of the $H_{\infty }$ norm by tuning gains, transfer functions, state space models etc. in the control loop. Therefore, state feedback control can be modified to achieve optimal and robust performance.

To determine whether this is indeed the case, we developed two novel state feedback controllers by supplementing the original state feedback gain with additional gains, thus forming two different versions of PI control. Owing to its simplicity and ease of implementation, PI/PID control is the most widely used control algorithm [71]. For the first controller, Single Input Single Output (SISO) PI controllers were added. In the second controller, a Multi-Input Multi-Output (MIMO) gain and integrator was added to form a MIMO PI controller. The looptune synthesized controllers were then compared against each other and the LQR synthesized single gain controller. As of present, no research article has compared the performance of looptune to LQR or other control techniques, especially in the context of position control for a 4-DOF robotic manipulator. The main contributions of this paper can be summarized as follows:

1. Development of two novel state feedback controllers by modifying the original single gain state feedback controller.
2. Tuning the gains of the novel state feedback controllers to achieve the desired specification.
3. Comparison of LQR and looptune synthesized controllers in terms of transient and steady-state performance, and robustness to uncertainty.
4. Comparison of two looptune synthesized controllers for trajectory tracking.
5. Using the results of the comparison to justify superiority of looptune over LQR in terms of performance and robustness.
6. Demonstrating the looptune synthesized second control system consisting of Single Input Single Output (SISO) Proportional Integral (PI) controllers and state feedback matrix as the candidate controller on a comparative basis.

The remainder of this paper is structured as follows. Section 2 describes the mathematical model of the robotic arm, while details on the performance specification, controller design and trajectory tracking are presented in Section 3. Simulation results are analyzed and discussed in Section 4. In Section 5, each of the controllers is analyzed for robustness under uncertainty. Lastly, Section 6 describes the controller selection in terms of the best overall performance and concludes the entire paper.

## 2 Model description

The robotic arm used for this research is a modification of an 8-DOF arm developed using SimMechanics in MATLAB 2012a for demonstration purposes by MathWorks. It originally consisted of five links namely the base, upper arm, forearm, wrist and gripper assembly. The base and upper arm were linked together using a 3-DOF spherical joint, while the upper arm-forearm, forearm-wrist, and wrist-gripper assembly were all linked together by 1-DOF revolute joints. Finally, the gripper assembly was composed of two fingers each having 1-DOF.

**Fig 1:**
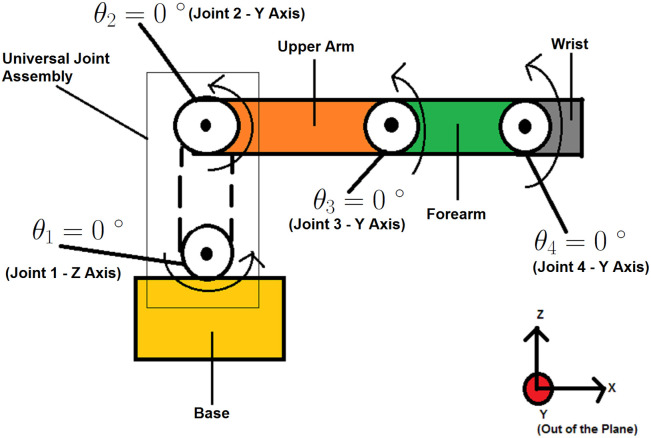
Home configuration of the robotic manipulator.

For our research, the gripper assembly was removed and the spherical joint was replaced by 2-DOF universal joint. The rest of the manipulator structure was kept intact. This modification resulted in a 4-DOF robotic manipulator which is more or less similar to many commercially available robotic manipulators. Fig 1 shows a basic sketch of the manipulator outlining its overall structure, home configuration and initial conditions. The initial conditions of the manipulator are given as *θ*_1_ = 0, *θ*_2_ = 0, *θ*_3_ = 0 and *θ*_4_ = 0.

**Fig 2:**
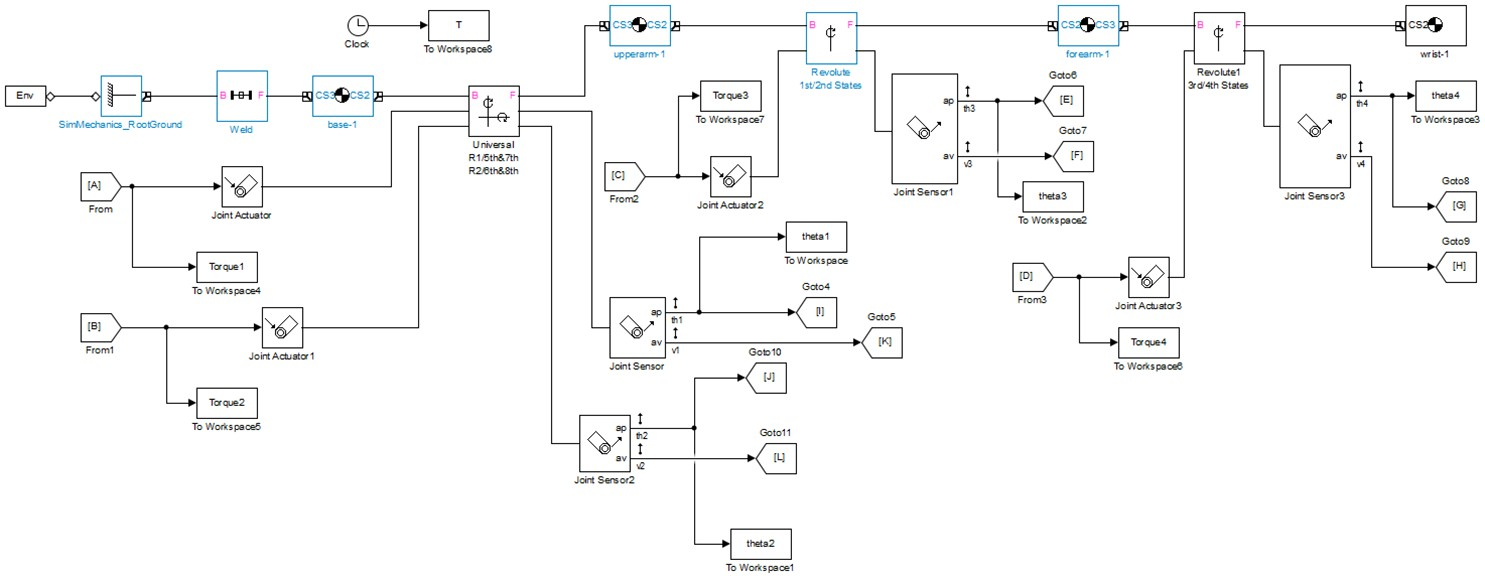
Simulink open-loop model of the robotic manipulator.

Fig 2 shows the implementation of the robotic arm in SimMechanics, a multibody toolbox in Simulink/MATLAB used for simulation and modeling of mechanical and electromechanical systems. The “Body Block” represents an individual robotic arm link such as the base, forearm, upper arm, and wrist. The “Universal/Revolute Joint Block” and “Weld Joint Block” determine rotation and fixation between two robotic arm links respectively. Actuation of the joints is provided by the “Joint Actuator Block”, while output from the joint is obtained using “Joint Sensor Block”. The “Ground Block”, is an immobile ground point relative to an absolute inertial reference frame. Finally, the “Environment Block” is used for configuration of the simulation. The simulations have been performed on an Intel Core i5-2450M CPU system with 8 GB RAM and 500 GB Hard Disk. Table 1 describes the physical parameters of the SimMechanics model of the Manipulator.

**Table 1:** Physical parameters of the SimMechanics/Simulink model.

Link	Mass(kg)	Length(m)	Moment of Inertia Tensor(*kg*.*m*^2^)
Base	9.465	0.1328	$\left[\begin{array}{ccc} 0.0198 & 0.0003 & 0.0000\\ 0.0003 & 0.0401 & 0.0000\\ 0.0000 & 0.0000 & 0.0274 \end{array}\right]$
Upper Arm	0.8016	0.1003	$\left[\begin{array}{ccc} 0.0007 & -0.0001 & 0.0000\\ -0.0001 & 0.0025 & 0.0000\\ 0.0000 & 0.0000 & 0.0024 \end{array}\right]$
Forearm	1.005	0.1092	$\left[\begin{array}{ccc} 0.0007 & -0.0001 & 0.0000\\ -0.0001 & 0.0025 & 0.0000\\ 0.0000 & 0.0000 & 0.0024 \end{array}\right]$
Wrist	0.1517	0.0305	$1\text{e}-4\times \left[\begin{array}{ccc} 0.2711 & -0.0000 & -0.0000\\ -0.0000 & 0.4630 & 0.0000\\ -0.0000 & 0.0000 & 0.3879 \end{array}\right]$

As seen in Fig 1, each joint has been assigned a number along with its respective angle. Each joint axis orientation is given in the adjacent parenthesis. Using MATLAB’s “Linear Analysis Toolbox”, the robotic manipulator is linearized about the initial conditions given in Fig 1, inputs A, B, C and D, and outputs E, F, G, H, I, J, K, and L given in Fig 2. This results in a linear state space model. The dynamic equation of motion for a robotic manipulator is given in (1).

$$\begin{array}{c} \tau =M\left(\theta \right)\left(\ddot{\theta }\right)+V\left(\theta ,\dot{\theta }\right)+G\left(\theta \right) \end{array}$$ (1)

*M*(*θ*) is a positive definite inertia matrix, $V(\theta ,\dot{\theta })$ is a vector representing centrifugal and Coriolis forces, *G*(*θ*) is a vector for denoting gravitational forces and is a vector representing actuator torques. Deriving the above equations manually using Euler-Lagrange or Euler-Newton methodology is a tedious and lengthy procedure. SimMechanics allows for simulation and linear analysis of the robot dynamics without the need to derive (1).

The system described above has 8 states, 4 inputs and 8 outputs. The joints have been indexed as *i* = 1, 2, 3, 4 according to the configuration shown in Fig 1. The variables *θ*_*i*_, ${\dot{\theta }}_{i}$, ${\ddot{\theta }}_{i}$ and *τ*_*i*_ represent the angular position/displacement, angular velocity, angular acceleration and applied torque/actuation of the *i*^*th*^ joint respectively.

## 3 Controller design

### 3.1 Linear quadratic regulator

The control design strategy has been formulated with the performance specification outlined in Table 2. Since our focus is on position control only, a system with Type I steady-state error is proposed. For Type I systems, the steady-state error for a step input (position constant) is zero, finite for a ramp input (velocity constant) and infinite for a parabolic input (acceleration constant) [72].

**Table 2:** Specification of the proposed control system.

S. No	Parameters	Allowed Limit
1	Rise Time	3 seconds
2	Settling Time	6 seconds
3	Steady-State Error	10%
4	Maximum Peak Torque	± 130 Nm
5	Overshoot and Undershoot	15%

The **Q** and **R** matrices are the state and control weighting matrices respectively and are shown below by (2) and (3):

$$\begin{array}{c} Q=[\begin{array}{cccccccc} \theta_{3} & 0 & 0 & 0 & 0 & 0 & 0 & 0\\ 0 & {\dot{\theta }}_{3} & 0 & 0 & 0 & 0 & 0 & 0\\ 0 & 0 & \theta_{4} & 0 & 0 & 0 & 0 & 0\\ 0 & 0 & 0 & {\dot{\theta }}_{4} & 0 & 0 & 0 & 0\\ 0 & 0 & 0 & 0 & \theta_{1} & 0 & 0 & 0\\ 0 & 0 & 0 & 0 & 0 & \theta_{2} & 0 & 0\\ 0 & 0 & 0 & 0 & 0 & 0 & {\dot{\theta }}_{1} & 0\\ 0 & 0 & 0 & 0 & 0 & 0 & 0 & {\dot{\theta }}_{2} \end{array}] \end{array}$$ (2)

$$\begin{array}{c} R=[\begin{array}{cccc} \tau_{1} & 0 & 0 & 0\\ 0 & \tau_{2} & 0 & 0\\ 0 & 0 & \tau_{3} & 0\\ 0 & 0 & 0 & \tau_{4} \end{array}] \end{array}$$ (3)

Eqs (2) and (3) are then used to solve the Algebraic Riccati Equation given as follows in (4):

$$\begin{array}{c} A^{T}X+XA-XBR^{-1}B^{T}X+Q=0 \end{array}$$ (4)

Where **A** represents the state matrix, **B** is the control input matrix, and **X** is a solution to the above equation. By substituting **X** into (5), the control law ***τ*** is obtained as under.

$$\begin{array}{c} \tau =-R^{-1}B^{T}Xx=-K_{QR}x \end{array}$$ (5)

Here ***K***_***LQR***_ represents the gain calculated using the LQR algorithm, while $x={\left[\theta_{3}\;{\dot{\theta }}_{3}\;\theta_{4}\;{\dot{\theta }}_{4}\;\theta_{1}\;\theta_{2}\;{\dot{\theta }}_{1}\;{\dot{\theta }}_{2}\right]}^{T}$ denotes the state vector of the system.

**Fig 3:**
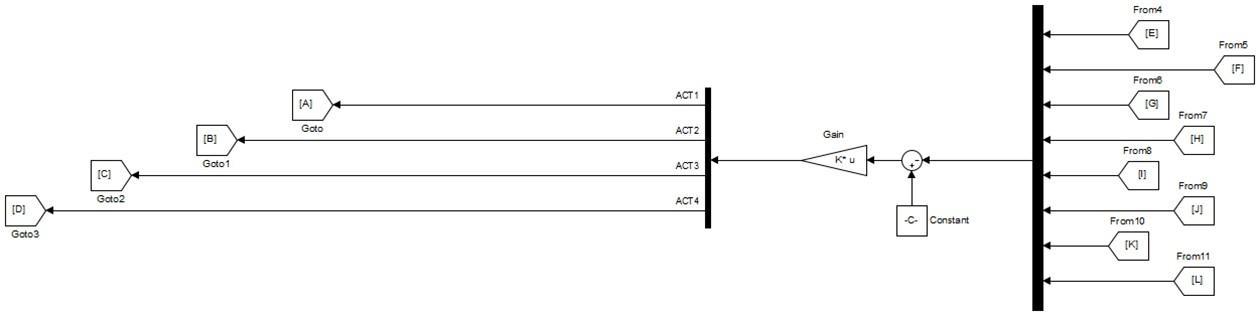
LQR state feedback control in Simulink.

Fig 3 shows the implementation of the control scheme in Simulink. Outputs from the sensor blocks are multiplexed into a single signal and then subtracted from the signal emanating from the reference “Constant Block”. The difference or error signal is then fed to state feedback gain represented by the “Gain Block”.

Next, a quantity $\tilde{x}$ is defined in (6) to denote the error or deviation from the reference or desired signal **r**. Using (6), closed loop dynamical model of the system is represented in (7).

$$\begin{array}{c} \tilde{x}=r-x \end{array}$$ (6)

$$\begin{array}{c} \tilde{\dot{x}}=\left({A-BK}_{LQR}\right)\tilde{x}-Ar \end{array}$$ (7)

### 3.2 Control cases

Using the idea presented in [73], two cases of initial and final positions have been considered to check whether controller performance is adversely affected by change of initial or final conditions. The first case represents the home configuration of the robotic arm, therefore the initial conditions in this configuration are zero by default. In the second set, all initial conditions have been set to 35 degrees. The initial condition for the second set is the final position of the first set, while the final position for the second set is obtained by adding 35 degrees. The joints of many commercially available robotic manipulators are capable of executing such a motion [74–76]. As shown in the following sections, each of the controllers is insensitive to change in initial and final conditions.

#### 3.2.1 Case-1

$$\theta_{INITIAL}=[\begin{array}{c} 0\\ 0\\ 0\\ 0 \end{array}]$$

$$\theta_{FINAL}=[\begin{array}{c} 35\\ 35\\ 35\\ 35 \end{array}]$$

#### 3.2.2 Case-2

$$\theta_{INITIAL}=[\begin{array}{c} 35\\ 35\\ 35\\ 35 \end{array}]$$

$$\theta_{FINAL}=[\begin{array}{c} 70\\ 70\\ 70\\ 70 \end{array}]$$

### 3.3 Manual tuning

In this procedure, the weights or values of *θ*_1_, *θ*_2_, *θ*_3_ and *θ*_4_ inside the cost matrix **Q** are adjusted while keeping the values of ${\dot{\theta }}_{1}$, ${\dot{\theta }}_{2}$, ${\dot{\theta }}_{3}$ and ${\dot{\theta }}_{4}$ constant at 1, until the performance objectives specified in Table 2 have been achieved. Likewise, the values of *τ*_1_, *τ*_2_, *τ*_3_ and *τ*_4_ inside the matrix **R** are kept constant at 1. The final **Q** and **R** matrices obtained are shown respectively in (8) and (9).

$$\begin{array}{c} Q=[\begin{array}{cccccccc} 10 & 0 & 0 & 0 & 0 & 0 & 0 & 0\\ 0 & 1 & 0 & 0 & 0 & 0 & 0 & 0\\ 0 & 0 & 10 & 0 & 0 & 0 & 0 & 0\\ 0 & 0 & 0 & 1 & 0 & 0 & 0 & 0\\ 0 & 0 & 0 & 0 & 10 & 0 & 0 & 0\\ 0 & 0 & 0 & 0 & 0 & 10 & 0 & 0\\ 0 & 0 & 0 & 0 & 0 & 0 & 1 & 0\\ 0 & 0 & 0 & 0 & 0 & 0 & 0 & 1 \end{array}] \end{array}$$ (8)

$$\begin{array}{c} R=[\begin{array}{cccc} 1 & 0 & 0 & 0\\ 0 & 1 & 0 & 0\\ 0 & 0 & 1 & 0\\ 0 & 0 & 0 & 1 \end{array}] \end{array}$$ (9)

Substituting (8) and (9) into (4), and then substituting the result into (5), gives the value of the state feedback gain shown by (10).

**Fig 4:**
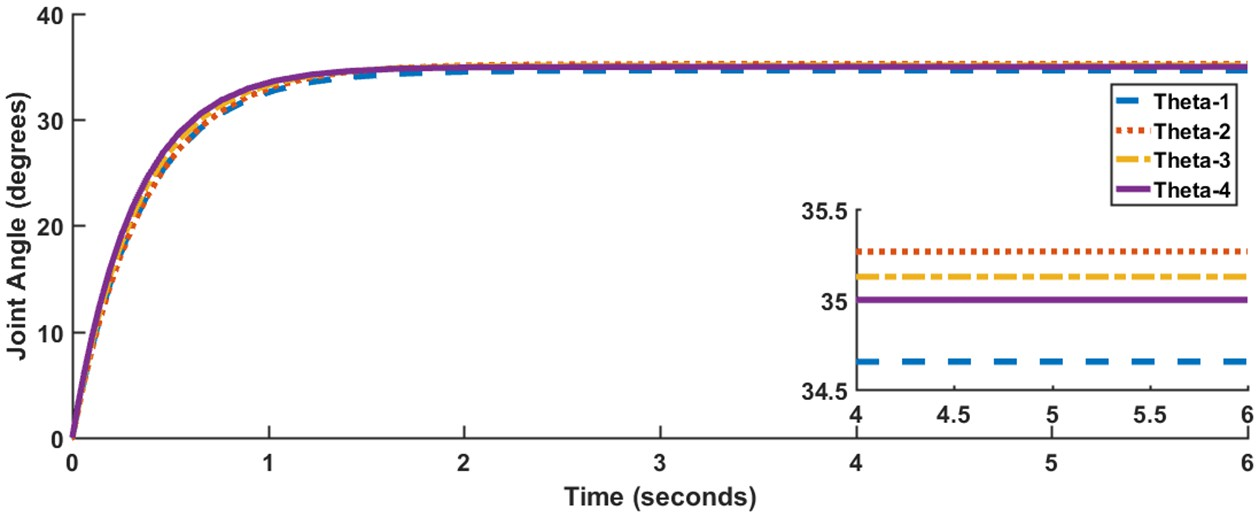
Response for LQR, Case I.

**Fig 5:**
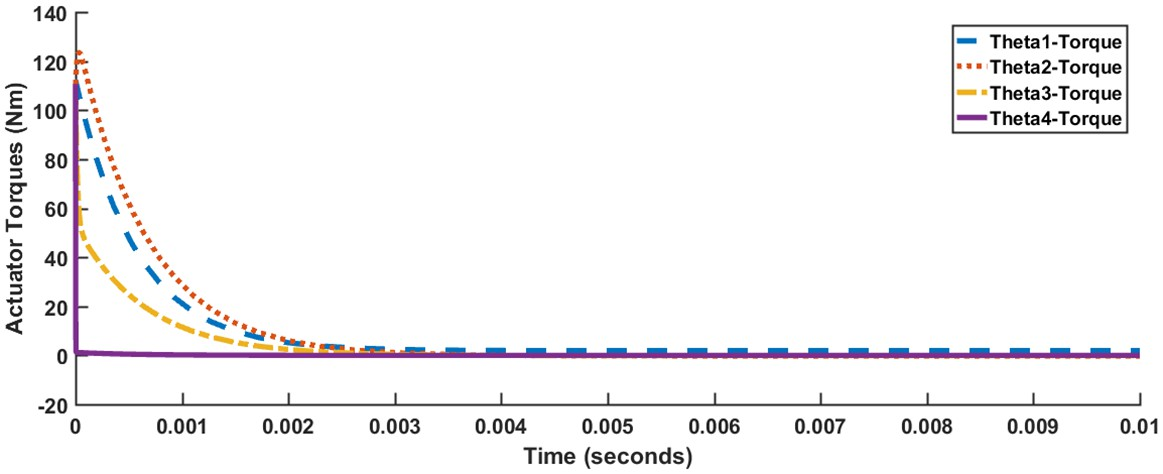
Actuator torques for LQR, Case I.

Fig 4 shows the response of all four joint angles, while Fig 5 shows the response of actuator torques. The timescale in Fig 5 has been compressed to 10 milliseconds for better visibility. The responses satisfy the performance specification criteria enlisted in Table 1. However, all of the joint angles excluding *θ*_4_ suffer from a slight steady-state error. Also, each of the actuator torque exceeds 100 Nm.

$$\begin{array}{c} K_{LQR}=[\begin{array}{cccccccc} 0.0000 & 0.0000 & 0.0000 & 0.0012 & 3.1736 & 0.0000 & 1.1091 & 0.0000\\ -0.0037 & 0.0422 & 0.0000 & 0.0000 & 0.0000 & 3.1596 & 0.0000 & 1.1084\\ 3.1584 & 1.0199 & 0.0000 & 0.0000 & -0.0000 & -0.0041 & 0.0000 & 0.0421\\ 0.0000 & -0.0000 & 3.1622 & 1.0002 & -0.0000 & -0.0000 & 0.0012 & -0.0000 \end{array}] \end{array}$$ (10)

Table 3 summarizes the results of the manually tuned LQR controller for Case I and Case II. The results of Case I and Case II are similar as far as the values of settling time, rise time and peak absolute are concerned, varying by 3% at the most. The values of overshoot, undershoot and steady-state error, although different, are negligible in comparison to the 15% and 10% requirements on steady-state error and percentage overshoot/undershoot respectively. Therefore, Control Case I and Case II can be assumed to be similar. It is also clearly evident from Table 3 that a prompt response is generated at a considerable cost of control energy. Despite expending such a large amount of control energy, the presence of steady-state error has not been eliminated.

**Table 3:** Summary of results for LQR.

Case	*θ*_*I*_	*θ*_*F*_	Overshoot (%)	Undershoot (%)	Settling Time (s)	Rise Time(s)	Peak Absolute Torque (Nm)	Steady-State Error (%)
I	0	35	0	0	1.3837	0.7821	111.7619	0.9866
	0	35	0	0.003265	1.4361	0.8190	123.5421	0.7568
	0	35	0	0	1.3328	0.7447	110.3999	0.3579
	0	35	0	0	1.2360	0.6978	110.6765	0.008096
II	35	70	0.0001391	0	1.3786	0.7724	111.4552	0.02858
	35	70	0.0001647	0	1.4275	0.8020	119.4106	0.7357
	35	70	0.00007223	0	1.3104	0.7403	110.3999	0.1973
	35	70	0.00002047	0	1.2463	0.6972	110.6761	0.005756

In Section 3.4, the state feedback matrix **K** will be augmented with 4 SISO PI controllers. The feedback gain and SISO PI controllers will be simultaneously tuned with looptune to remove the steady-state error and keep the actuator torques below 100 Nm.

### 3.4 Structured $H_{\infty }$ based looptune

The looptune command converts the user specified requirements of control bandwidth, rise time, settling time etc. into weighting functions. These functions are in turn translated into an $H_{\infty }$ optimization problem. Looptune then invokes systune to minimize the $H_{\infty }$ norm by tuning user specified parameters or objects in the control loop such as gains, transfer functions, state space models, etc.

To achieve the desired bandwidth, looptune tunes the parameters such that the open-loop gain crosses 0 dB in the crossover frequency interval specified by the user. Similarly, the open-loop response is shaped to perform integral action at low frequencies. Finally, the high-frequency roll-off exceeding -20 dB/decade guarantees robustness.

After successive trial and error, the gain crossover band is selected at 0.1 ≤ *ω* ≤ 100000 rad/s. The control system response time and bandwidth are also determined in this particular region.

#### 3.4.1 State feedback gain augmented with SISO PI controllers (SFG-SISO PI)

**Fig 6:**
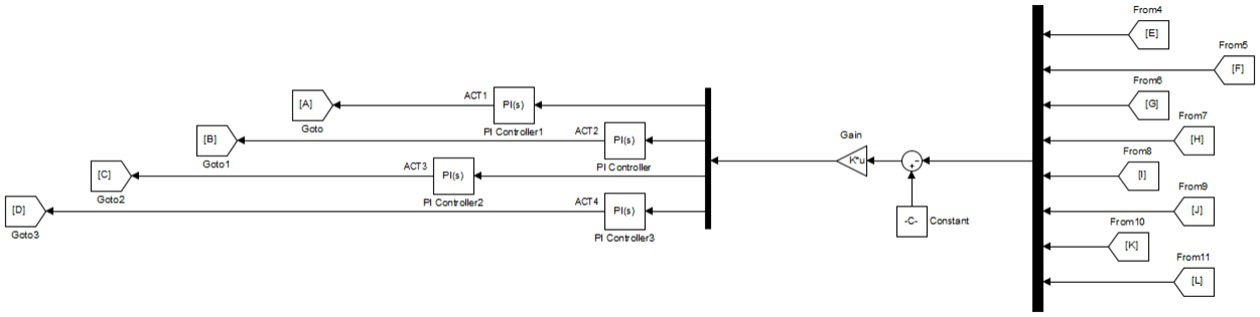
SFG-SISO PI control in Simulink.

To improve the transient and steady-state performance observed in Table 3, the feedback loop is modified to include scalar 1 × 1 PI regulators. As depicted in Fig 6, a set of 4 SISO PI regulators is used, whereby each regulator is placed in series with each of the 4 actuators.

The looptune command is then applied to simultaneously find optimal values for the gains and **K**. Eqs (11) and (12) represent the control law and closed loop error dynamics for the second control system respectively. The gains *k*_*P*_ and *k*_*I*_ respectively denote the proportional and integral gains. The proportional and integral gains are enclosed in 4 × 4 matrices ***K***_***P−SISO***_ and ***K***_***I−SISO***_, represented by (13) and (14) respectively.

$$\begin{array}{c} \tau =K_{P-SISO}K\tilde{x}\left(t\right)+K_{I-SISO}K\int_{-\infty }^{t}\tilde{x}\left(\alpha \right)d\alpha \end{array}$$ (11)

$$\begin{array}{c} \tilde{\dot{x}}=\left(A-BK_{P-SISO}K\right)\tilde{x}\left(t\right)-BK_{I-SISO}K\int_{-\infty }^{t}\tilde{x}\left(\alpha \right)d\alpha -Ar \end{array}$$ (12)

$$\begin{array}{c} K_{P-SISO}=[\begin{array}{cccc} k_{P1} & 0 & 0 & 0\\ 0 & k_{P2} & 0 & 0\\ 0 & 0 & k_{P3} & 0\\ 0 & 0 & 0 & k_{P4} \end{array}] \end{array}$$ (13)

$$\begin{array}{c} K_{I-SISO}=[\begin{array}{cccc} k_{I1} & 0 & 0 & 0\\ 0 & k_{I2} & 0 & 0\\ 0 & 0 & k_{I3} & 0\\ 0 & 0 & 0 & k_{I4} \end{array}] \end{array}$$ (14)

Since looptune requires an initial value for all of the tunable parameters present in the control loop, ***K***_***LQR***_ in (10) will be used to initialize looptune for tuning **K**. The initial values for the proportional and integral gains are given by (15) and (16).

$$\begin{array}{c} K_{P-SISO-INIT.}=[\begin{array}{cccc} 1 & 0 & 0 & 0\\ 0 & 1 & 0 & 0\\ 0 & 0 & 1 & 0\\ 0 & 0 & 0 & 1 \end{array}] \end{array}$$ (15)

$$\begin{array}{c} K_{I-SISO-INIT.}=[\begin{array}{cccc} 1 & 0 & 0 & 0\\ 0 & 1 & 0 & 0\\ 0 & 0 & 1 & 0\\ 0 & 0 & 0 & 1 \end{array}] \end{array}$$ (16)

At the end of the tuning process the final values for **K**, ***K***_***P−SISO***_ and ***K***_***I−SISO***_ are given as follows in (17), (18) and (19) respectively:

$$\begin{array}{c} K_{FINAL}=[\begin{array}{cccccccc} 0.0002 & -0.0015 & 0.0123 & 0.0698 & 2.8525 & -0.0000 & 1.5843 & 0.0004\\ -0.0453 & 0.0215 & -0.0001 & 0.0068 & -0.0002 & 3.0005 & -0.0009 & 1.1751\\ 3.0099 & 1.0951 & 0.0001 & -0.0061 & 0.0016 & -0.0399 & -0.0004 & 0.0160\\ 0.0085 & -0.0173 & 2.8283 & 0.0423 & 0.0320 & 0.0007 & 0.0306 & -0.0002 \end{array}] \end{array}$$ (17)

$$\begin{array}{c} K_{P-SISO-FINAL}=[\begin{array}{cccc} 0.4695 & 0 & 0 & 0\\ 0 & 0.6536 & 0 & 0\\ 0 & 0 & 0.6597 & 0\\ 0 & 0 & 0 & 0.2564 \end{array}] \end{array}$$ (18)

$$\begin{array}{c} K_{I-SISO-FINAL}=[\begin{array}{cccc} 0.9991 & 0 & 0 & 0\\ 0 & 0.9999 & 0 & 0\\ 0 & 0 & 1.0000 & 0\\ 0 & 0 & 0 & 1.0000 \end{array}] \end{array}$$ (19)

**Fig 7:**
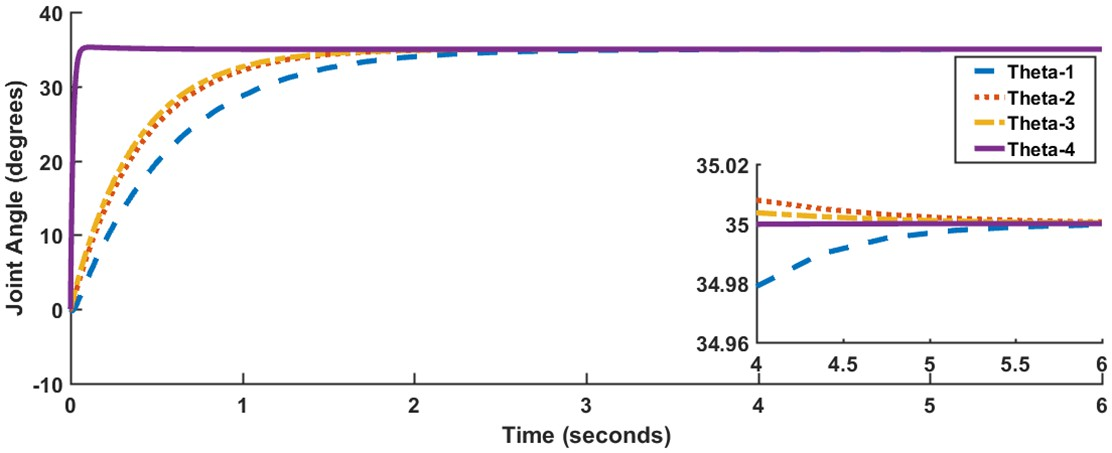
SFG-SISO PI response, Case I.

**Fig 8:**
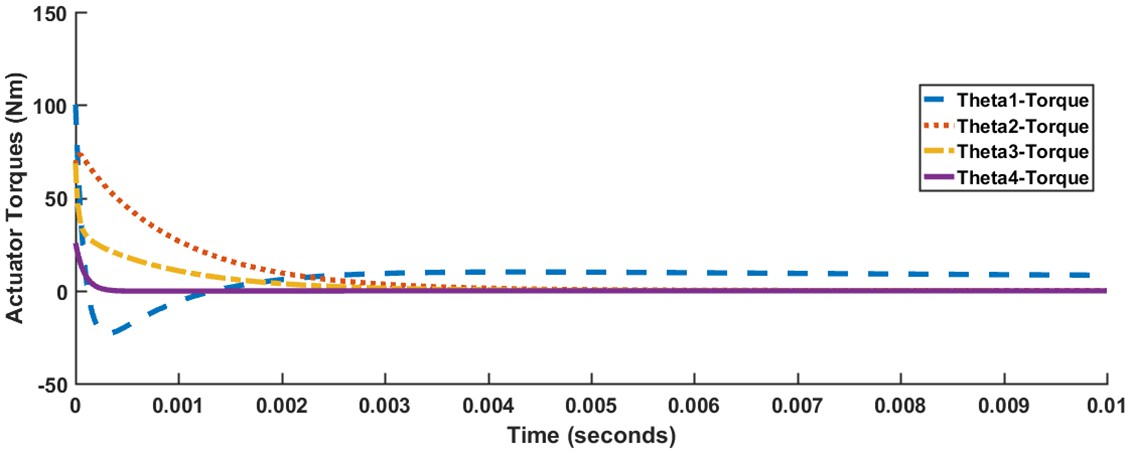
SFG-SISO PI actuator response, Case I.

Joint angle responses are shown in Fig 7, while Fig 8 shows actuator torques. Since the response and actuator torque profiles of Case I and Case II are similar, only the plots of Case I have been shown. It is evident from both Figs 7 and 8 that not only has the steady-state error been removed but also less control effort is expended. The degradation in transient parameters such as rise time and settling appears to be negligible for angles, *θ*_2_ and *θ*_3_ while there is considerable improvement in the response of *θ*_4_. Table 4 summarizes the results for SFG-SISO PI.

**Table 4:** Summary of results for SFG-SISO PI controllers.

Case	*θ*_*I*_	*θ*_*F*_	Overshoot (%)	Undershoot (%)	Settling Time (s)	Rise Time (s)	Peak Absolute Torque (Nm)	Steady-State Error (%)
I	0	35	0	0.5316	2.1867	1.2366	100.2736	0
	0	35	0.04469	0.007096	1.5051	0.8727	73.2847	0
	0	35	0.01779	0	1.4364	0.8143	68.6127	0
	0	35	0.7894	0	0.05053	0.03026	25.7508	0
II	35	70	0	0	2.1734	1.2111	100.2736	0
	35	70	0.01510	0	1.4748	0.8522	68.2612	0
	35	70	0.0003536	0	1.441	0.8122	68.6127	0
	35	70	0.3873	0	0.05069	0.03029	25.7508	0

Similar to Table 3, the values of Case I and Case II in Table 4 are similar in terms of settling time, rise time, peak absolute torque, and steady-state error, with a maximum variation of 7% in peak absolute torque for joint angle *θ*_2_. Again, the difference in values of overshoot and undershoot are insignificant when compared with the requirement of 15%.

#### 3.4.2 State feedback gain augmented with MIMO integral gain (SFG-MIMO PI)

For the third control system, a single 4 × 8 matrix ***K***_***I***_ is added in the feedback loop and cascaded with an integrator. The output from the integrator block is summed with the output from the state feedback matrix ***K***_***P***_.

**Fig 9:**
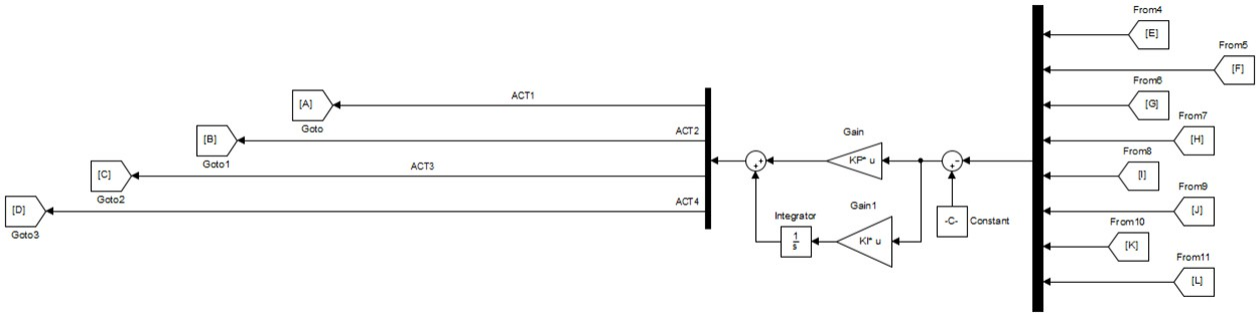
SFG-MIMO PI control in Simulink.

The main difference from the second control system lies in the fact that both ***K***_***I***_ and ***K***_***P***_ are 4 × 8 matrix gains as opposed to 1 × 1 scalar *k*_*P*_ and *k*_*I*_ gains. The gain ***K***_***P***_ is simultaneously acting as a state feedback gain as well as proportional gain in the third control system. On the other hand, the state feedback gain **K** in SFG-SISO PI is separate from the proportional gain *k*_*P*_. The implementation of this scheme is shown in Fig 9.

Here ***K***_***P***_ is obtained by tuning ***K***_***LQR***_ computed in (10). This results in a MIMO PI controller. Eqs (20) and (21) describe the dynamics of this controller.

$$\begin{array}{c} \tau =K_{P}\tilde{x}\left(t\right)+K_{I}\int_{-\infty }^{t}\tilde{x}\left(\alpha \right)d\alpha \end{array}$$ (20)

$$\begin{array}{c} \tilde{\dot{x}}=\left(A-BK_{p}\right)\tilde{x}\left(t\right)-BK_{I}\int_{-\infty }^{t}\tilde{x}\left(\alpha \right)d\alpha -Ar \end{array}$$ (21)

The integral gain ***K***_***I***_ will be initialized by the value given in (22). The final tuned values of ***K***_***P***_ and ***K***_***I***_ are shown in (23) and (24) respectively. Figs 6 and 7 show the response for joint angles and the actuator torques for Case I respectively.

$$\begin{array}{c} K_{I-INITIAL}=[\begin{array}{cccccccc} 2 & 2 & 2 & 2 & 2 & 2 & 2 & 2\\ 2 & 2 & 2 & 2 & 2 & 2 & 2 & 2\\ 2 & 2 & 2 & 2 & 2 & 2 & 2 & 2\\ 2 & 2 & 2 & 2 & 2 & 2 & 2 & 2 \end{array}] \end{array}$$ (22)

$$\begin{array}{c} K_{P-\text{FINAL}}=[\begin{array}{cccccccc} -0.0387 & -0.1626 & -0.2321 & 0.0152 & 2.7122 & -0.0327 & 1.2367 & -0.1113\\ -0.0429 & -0.0034 & -0.0751 & 0.0917 & -0.1372 & 3.0417 & 0.0582 & 1.2559\\ 3.0082 & 1.2135 & -0.0831 & 0.0397 & -0.1162 & -0.0205 & -0.0010 & -0.0671\\ 0.0163 & 0.0076 & 2.8202 & 0.0882 & 0.0046 & 0.0947 & -0.0095 & -0.0130 \end{array}] \end{array}$$ (23)

$$\begin{array}{c} K_{I-\text{FINAL}}=[\begin{array}{cccccccc} 1.1252 & 2.0603 & 1.1471 & 1.8759 & 4.3302 & 1.1130 & 2.4178 & 2.0772\\ 1.1307 & 2.0609 & 1.1362 & 1.9844 & 1.1922 & 4.4679 & 1.9819 & 2.1491\\ 4.4781 & 2.1191 & 1.1280 & 2.0377 & 1.1739 & 1.1227 & 1.9686 & 2.0709\\ 1.5491 & 1.5771 & 4.7664 & 1.3251 & 1.5324 & 1.5733 & 1.4617 & 1.5500 \end{array}] \end{array}$$ (24)

**Fig 10:**
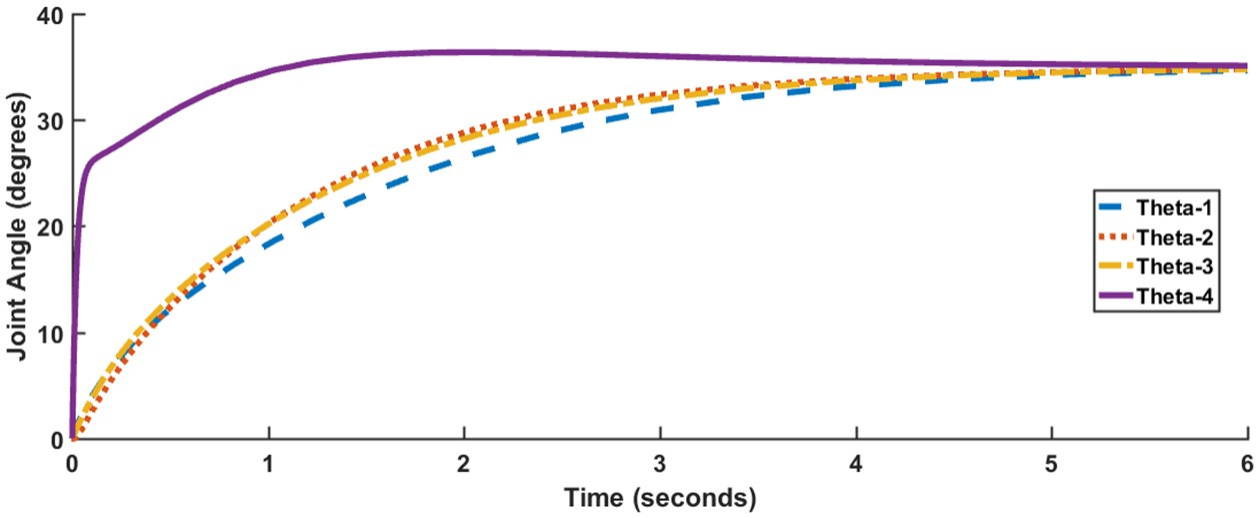
SFG-MIMO PI response, Case I.

**Fig 11:**
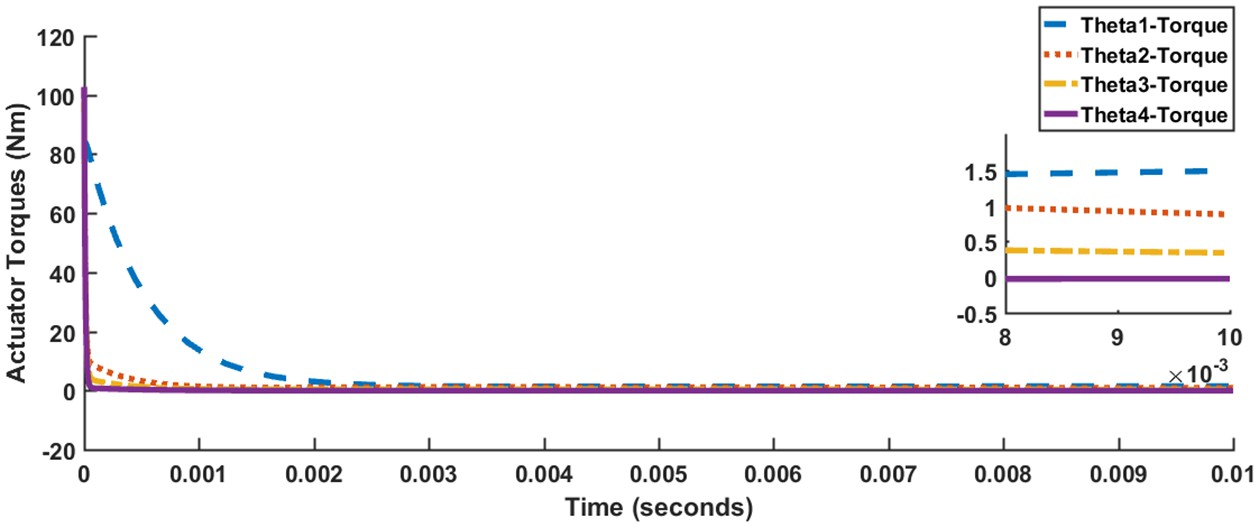
SFG-MIMO PI actuator response, Case I.

It is evident from Figs 10 and 11 that in comparison to both LQR and SFG-SISO PI, the SFG-MIMO PI controller is slow. Except for angle *θ*_1_, actuator effort for SFG-MIMO PI is greater than SFG-SISO PI. However, actuator effort for SFG-MIMO PI is still less than LQR for all joint angles. Numerical results for SFG-MIMO PI are presented in Table 5.

**Table 5:** Summary of results for SFG-MIMO PI.

Case	*θ*_*I*_	*θ*_*F*_	Overshoot (%)	Undershoot (%)	Settling Time (s)	Rise Time (s)	Peak Absolute Torque (Nm)	Steady-State Error (%)
I	0	35	0.001490	0.001416	5.1059	3.112089	84.3345	0.0002190
	0	35	0.0003520	0.08442	4.5631	2.51783	97.5285	0.00005200
	0	35	0.0003090	0	4.6920	2.712955	97.5978	0.00005370
	0	35	3.9675	0	3.6240	0.577677	102.7510	0.00003490
II	35	70	0.0001860	0	5.1547	3.0930	84.3043	0
	35	70	0.0002980	0	4.6126	2.5325	97.5285	0
	35	70	0.00008620	0	4.7081	2.7163	97.5978	0
	35	70	1.9562	0	3.6394	0.5902	102.7510	0

As discussed previously, the values of Case I and Case II are similar in terms of settling time, rise time and peak absolute torque, with maximum variation being 2%. When considering the requirement of Table 2, the differences in overshoot and undershoot are negligible for all the joint angles except for *θ*_4_. The values of overshoot observed for joint angle *θ*_4_, although different is nonetheless comparable.

### 5 Trajectory tracking control

**Fig 12:**
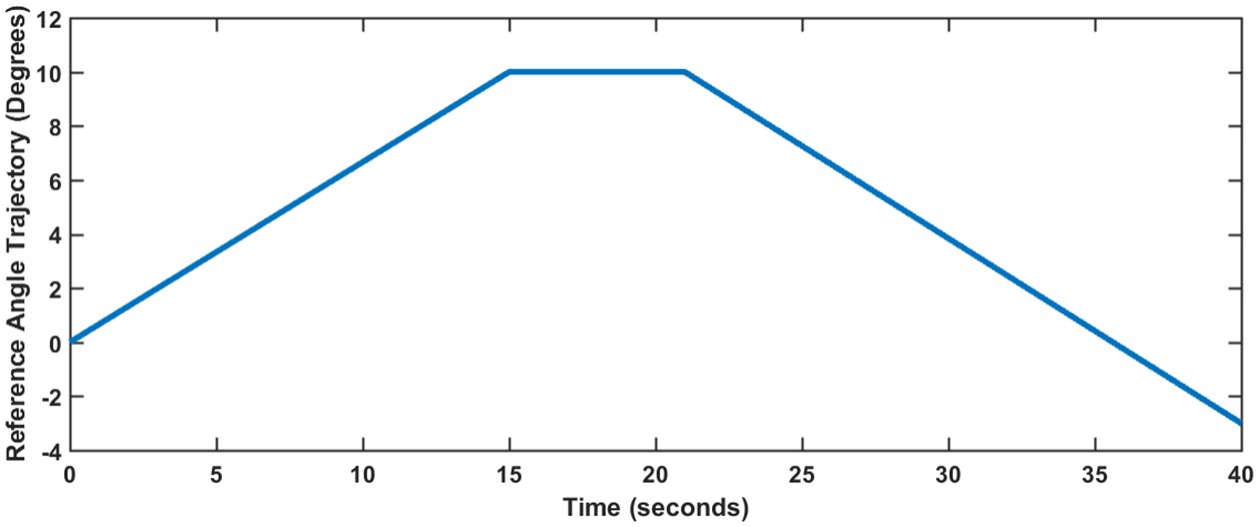
Reference trajectory for the robotic manipulator.

**Fig 13:**
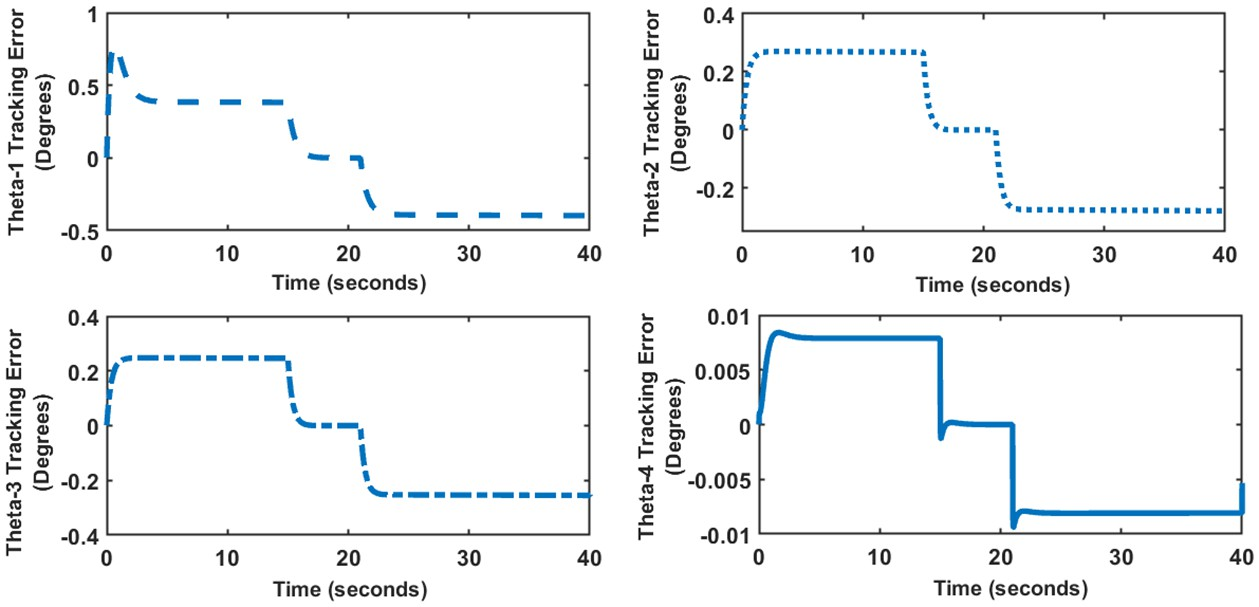
Tracking error for SFG-SISO PI control.

**Fig 14:**
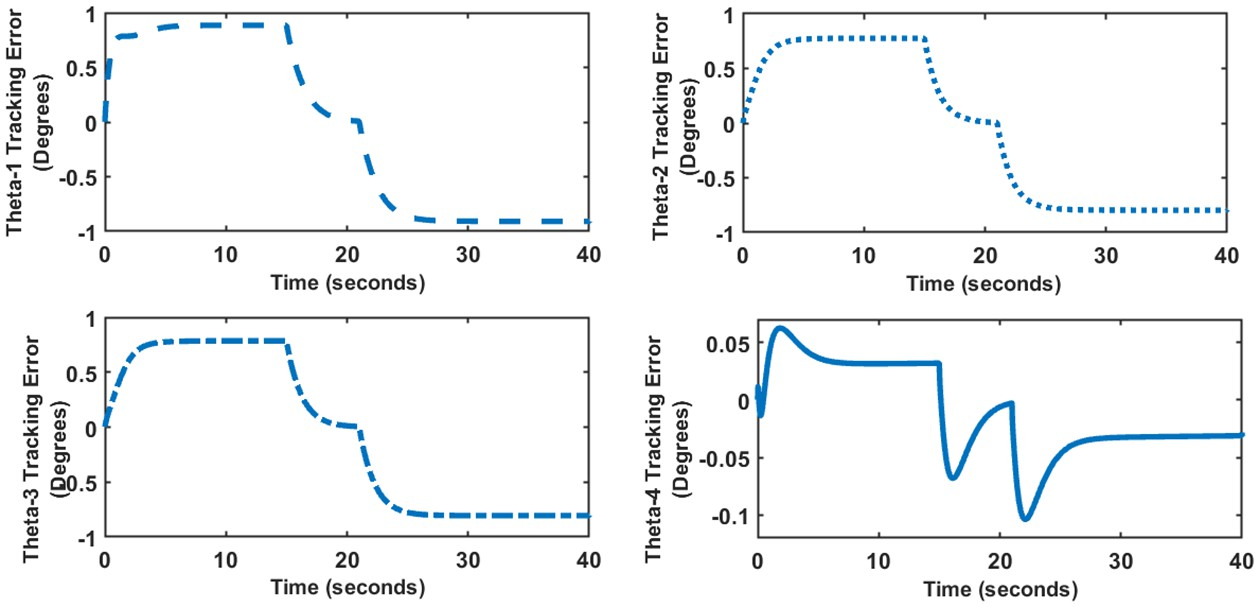
Tracking error for SFG-MIMO PI control.

Robotic manipulators perform pick and place tasks as one of their primary functions. In such cases, the manipulator has to follow a predefined time dependent trajectory as shown in Fig 12. Figs 13 and 14 show the trajectory tracking error for SFG-SISO PI and SFG-MIMO PI schemes respectively. The tracking error shown is the numerical difference between the input reference and the actual output response.

For both controllers, a finite steady-state error is observed during the rise and fall parts of the trajectory, which is within the ±10% limit given in Table 2. The steady-state error converges to zero as soon as the trajectory attains a constant value. This result is consistent with the expected behavior of a Type I control system. It is evident from both Figs 13 and 14 that SFG-SISO PI control has a comparatively smaller tracking error for all of the joint angles. In both cases, however, tracking error is the largest for the angle *θ*_1_, followed jointly by the angles *θ*_2_ and *θ*_3_. Remarkably, *θ*_4_ shows minimum tracking error.

## 4 Analysis of results

The results for the three control schemes are now compared in Table 6. The results are compared in terms of percentage reduction achieved with respect to the performance benchmarks specified in Table 2. Only the results for Case I are discussed since the results of Case II are more or less similar in terms of settling time, rise time and peak absolute torque for all three of the controllers as discussed above.

**Table 6:** Comparison of results in terms of percentage reduction.

Angle	Control Scheme	Overshoot Reduction %	Undershoot Reduction %	Settling Time Reduction %	Rise Time Reduction %	Peak Absolute Torque Reduction %	Steady-State Error Reduction %
*θ*_1_	LQR	100	100	77	74	14	90
	SFG-SISO PI	100	96	64	59	23	100
	SFG-MIMO PI	100	100	15	4	35	100
*θ*_2_	LQR	100	100	76	73	5	92
	SFG-SISO PI	100	100	75	71	44	100
	SFG-MIMO PI	100	99	24	16	25	100
*θ*_3_	LQR	100	100	78	75	15	96
	SFG-SISO PI	100	100	76	73	47	100
	SFG-MIMO PI	100	100	22	10	25	100
*θ*_4_	LQR	100	100	79	77	15	100
	SFG-SISO PI	95	100	99	99	80	100
	SFG-MIMO PI	74	100	40	81	21	100

It is apparent from Table 6 that there is a certain tradeoff involved between improvement in performance and controller effort. For example, the peak torque reduction for joint angle *θ*_1_ in SFG-SISO PI control is only 9% more than the peak torque reduction for LQR. Correspondingly, the reduction in the rise time and settling times is 15% and 13% lower in SFG-SISO PI as compared to LQR control. Remarkably, the reduction in peak actuator torque of by 44% and 47% in SFG-SISO PI for joint angles *θ*_2_ and *θ*_3_, gives nearly the same reduction in settling time and rise time as LQR control. For angle *θ*_4_ however, an 80% reduction in peak absolute torque brings about 99% reduction in rise time and settling time, which is truly remarkable.

For SFG-MIMO PI control, the peak torque reduction *θ*_1_ is 21% greater when compared to LQR. However, the reduction in rise and settling times is 70% and 62% less respectively. Similarly, for joint angle *θ*_2_ a reduction of 16% and 24% is respectively observed in rise time and settling time for a 25% decrease in actuator torque. Results of the joint angle *θ*_3_ are more or less similar to the angle *θ*_2_. In *θ*_4_, the rise time reduction is 4% greater than the corresponding value in LQR control but the settling time reduction is 39% less for a 21% reduction in actuator torque. In light of the above results, the SFG-SISO PI control is a better option compared to both LQR and SFG-MIMO PI in terms of tradeoff or balance between controller effort and performance.

## 5 Uncertainty analysis

Since no mathematical model of any physical system can be fully accurate, there will always be an element of uncertainty in any control system design. One of the ways in which the effect of uncertainty in robotic manipulators can be observed is to affect parametric changes in the manipulator system. This is done by increasing the mass of all individual links simultaneously by 3, 5, 7 and 9 times and observing the transient and steady-state performance.

**Fig 15:**
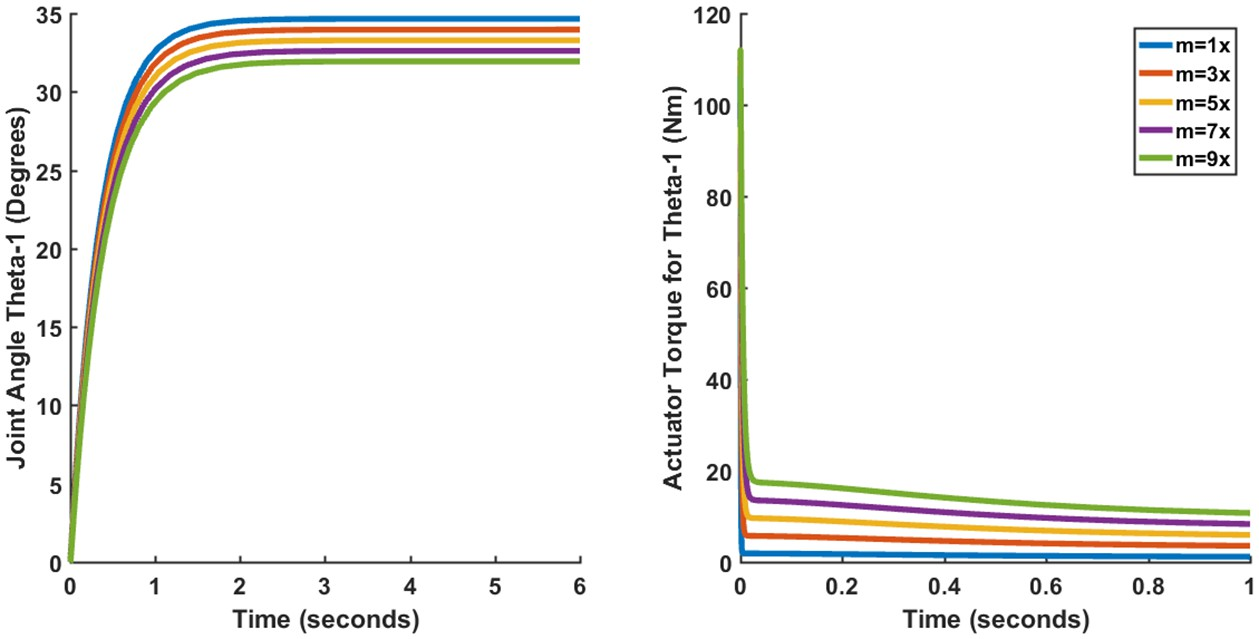
Performance of LQR (Case I) under mass variation.

**Fig 16:**
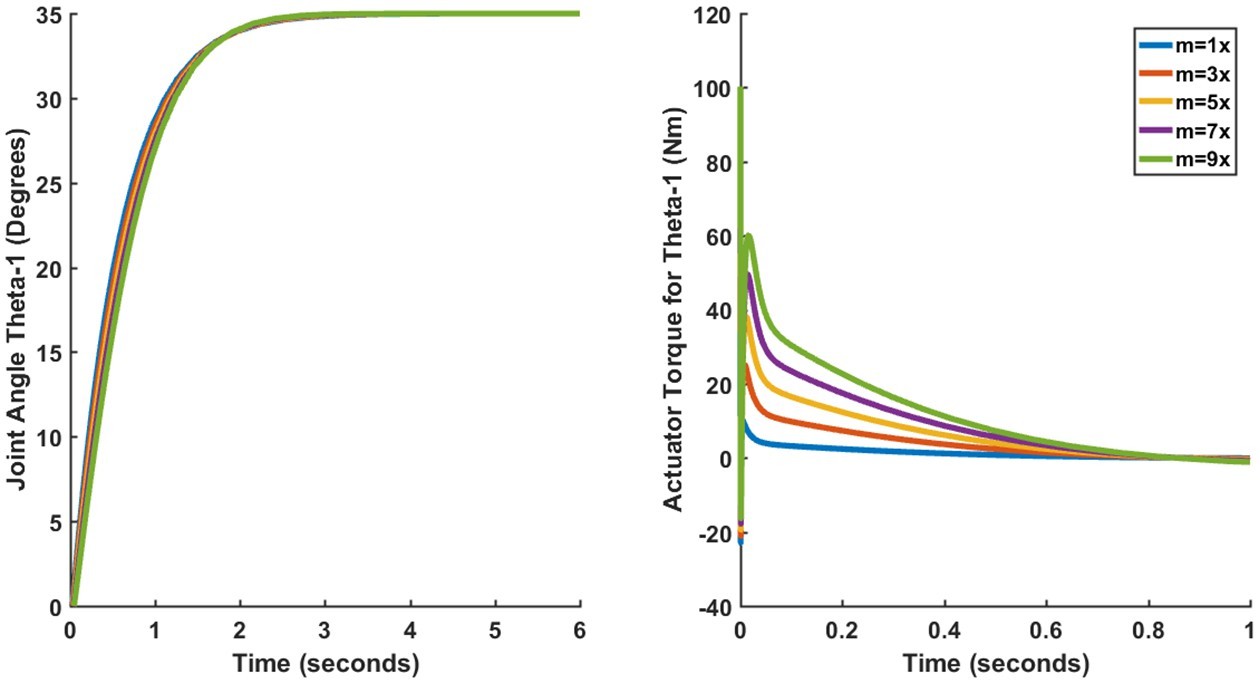
Performance of SFG-SISO (Case I) under mass variation.

**Fig 17:**
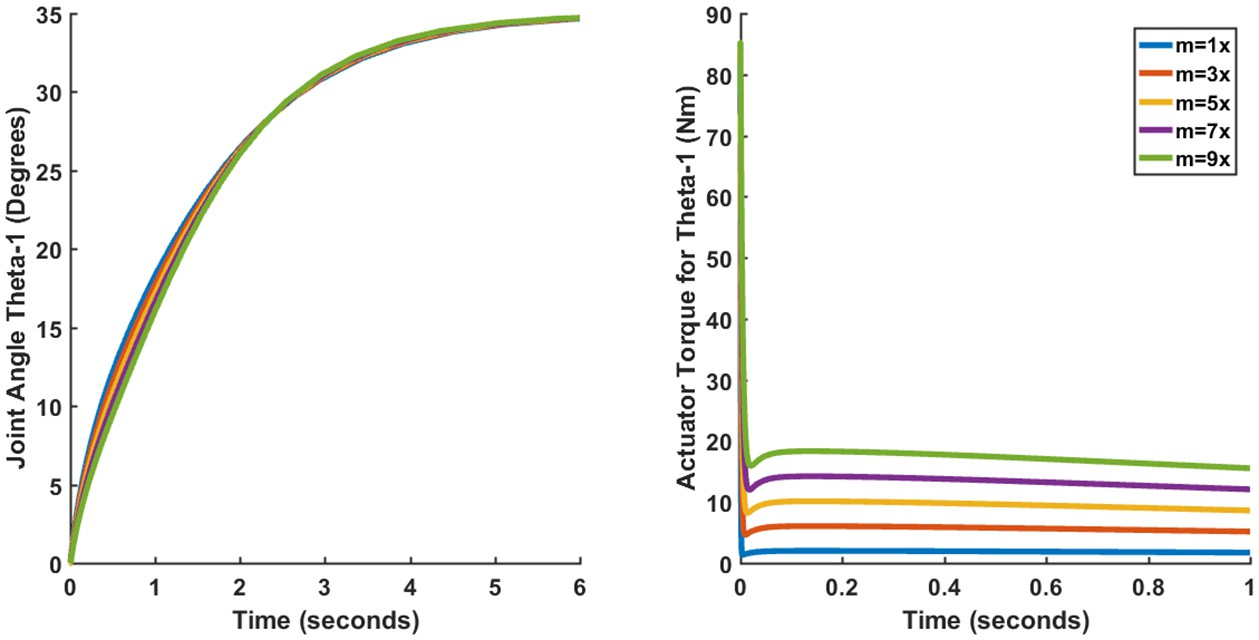
Performance of SFG-MIMO (Case I) under mass variation.

Only the results of the joint angle *θ*_1_ (Case I) have been shown since the plots of other joint angles are more or less the same. Figs 15–17 show the results for LQR, SFG-SISO PI and SFG-MIMO PI under mass variation respectively. It can be observed from Fig 15 that the steady-error increases in proportion to the increase in mass. Thus, it can be concluded that LQR control lacks robustness. Figs 16 and 17 on the other hand do not show appreciable deviation from the results presented earlier in Section 3.4. Therefore, both SFG-SISO PI and SFG-MIMO PI controllers show robustness to uncertainty. The only difference discernable is that the actuator torque takes a longer time to converge as the mass of each link increases. Nevertheless, there is little or no difference in peak actuator torque.

## 6 Conclusion

LQR and looptune were compared in terms of transient and steady-state performance, and robustness to uncertainty using a 4-DOF robotic arm. For this purpose, three different full state feedback control architectures were developed. The feedback gain for the first control system was computed using LQR. In comparison, SFG-SISO PI and SFG-MIMO PI controllers were synthesized by looptune. The SFG-SISO PI and SFG-MIMO PI controllers were also compared in terms of trajectory tracking. The value of the state feedback gain calculated using LQR was used as an initial value by looptune. Despite expending a large amount of control effort, the LQR was unable to eliminate steady-state error. Moreover, it was found to lack robustness against increasing mass variation. The SFG-SISO PI controller delivered a slightly slow response by considerably reducing its control effort. It also eliminated the steady-state error while showing robustness to uncertainty. For a slight decrease in control effort, the response of SFG-MIMO PI controller was slowest. However, it still showed robustness to uncertainty. Therefore, from an overall perspective, the SFG-SISO PI controller fares as the best controller, validates the superiority of looptune over LQR and affirms the potential of looptune for future control system applications.

In this research, we have proposed a Type-I (position control) system for a rigid robotic arm. The proposed methodology may be extended to include velocity or acceleration control. This can prove highly useful in the context of robotic automation processes such as welding, painting, packaging, pick and drop etc. In addition, it has been assumed that all of the states are measurable. Practically, one or more of the states may not be measurable, necessitating the requirement of an observer. Consequently, the performance of a Linear Quadratic Gaussian (LQG) (or other observer based controllers) may be compared against looptune synthesized observer based controllers. Likewise, looptune synthesized controllers can also be compared against adaptive or intelligent controllers discussed in Section 1. The performance or robustness of looptune can also be compared against manually tuned $H_{2}$ and $H_{\infty }$ controllers. Finally, the performance of looptune can be evaluated for flexible robotic manipulator models. In summary, there are multiple directions in which the methodology applied in this paper can be explored or further improved.
